# A parallel method for accelerating visualization and interactivity for vector tiles

**DOI:** 10.1371/journal.pone.0221075

**Published:** 2019-08-15

**Authors:** Wei Hu, Lin Li, Chao Wu, Hang Zhang, Haihong Zhu

**Affiliations:** 1 School of Resource and Environment Sciences, Wuhan University, Wuhan, China; 2 System Research Department, The 14th Research Institute of China Electronics Technology Group Corporation, Nanjing, China; 3 Geo Spatial Information Science Collaborative Innovation Center of Wuhan University, Wuhan University, Wuhan, China; 4 School of Geographic and Biologic Information, Nanjing University of Posts and Telecommunications, Nanjing, China; 5 Smart Health Big Data Analysis and Location Services Engineering Lab of Jiangsu Province, Nanjing University of Posts and Telecommunications, Nanjing, China; China University of Geosciences, CHINA

## Abstract

Vector tile technology is developing rapidly and has received increasing attention in recent years. Compared to the raster tile, the vector tile has shown incomparable advantages, such as flexible map styles, suitability for high-resolution screens and ease of interaction. Recent studies on vector tiles have mostly focused on improving the efficiency on the server side and have overlooked the efficiency on the client side, which affects user experience. Parallel computing provides solutions to this issue. Parallel visualization of vector tiles is a typical example of embarrassing parallelism; thus, estimating the computing times of each tile accurately and decomposing the workload into multiple computing units evenly are key to the parallel visualization of vector tiles. This article adopts mainstream parallel computing and proposes an efficient tile-based parallel method for accelerating geographical feature visualization by building computational weight functions (CWFs) of geographical feature visualizations. The computing time of each vector tile is estimated by the CWF, and an effective workload decomposition strategy is proposed such that the efficiency of vector tile visualization is improved on the client side. Furthermore, a tile-based reconstruction scheme for geographical features is also proposed. Experiments show that the R-squared value of the estimated computing times of vector tiles is 0.914 and that the computational efficiency of the parallel visualization of vector tiles with the proposed workload decomposition strategy is 18.6% higher than that of common parallel visualization. Finally, users can obtain the entire set of features effectively and accurately based on the proposed reconstruction scheme.

## Introduction

Vector tiles are an emerging scheme and technology for base maps, providing distinct merits in terms of interactivity compared with raster tiles. Like the raster tile map, the vector tile map is divided into small pieces called “tiles” that are transmitted to the client side according to the requested region [1]. However, the vector tiles used for rendering on the client or web service side in WebGIS are quite different from the raster tiles. Essentially, raster tiles deliver to the client images that have been pre-rendered and stored on the server, while vector tiles store the vector forms of geographic features that can be abstracted as points, lines and polygons [2] and render the features in map symbolization on the client side.

Although raster tiles have been used for over a decade and are widely adopted by the geospatial community, they still have some disadvantages, including poor adaptability, inflexible resolution and style and lack of interactivity. The emergence of vector tiles is significant and crucial for compensating for the deficiencies of raster tiles [3]. First, vector tile rendering is completed on the client side and affords great adaptability for users in terms of map styles customization. Second, vector tiles can be rendered at any display device resolution to achieve high resolution. A vector tile is usually smaller than a raster tile at the same resolution, which means that vector tiles can reduce the cost of data storage and transmission [2]. Furthermore, each feature contained in a tile has attribute data and geometry data, and users can access the actual features for information inquiry and spatial analysis on the client side, even in disconnected environments or environments with limited network connectivity [1,2,4]. Finally, as is often the case in the field of information technology (IT), vector tiles exhibit the best performance on machines with newer hardware. Due to these advantages, vector tile technology is developing rapidly and has received increasing attention in recent years [1–7].

Most studies have focused on the efficiency of vector tiles on the server side. Antoniou et al. [4] proposed a new method for vector data transmission over the web using tile technology, and the experimental results show that tile technology is an effective method of vector data transmission. Shang [1] explored the transmission efficiency of three vector tile encoding formats: GeoJSON, TopoJSON and Google Protocol Buffers, and indicated that the proposed solution improves the application performance. Wan et al. [6] proposed a flexible storage framework that provides feasible methods for tiled map data parallel clipping and retrieval operations within a distributed NoSQL database environment on servers. The experiments show that the NoSQL-based parallel tile management framework can support applications with enormous volumes of vector tile data and can improve the performance of the tiled map service. Eugene et al. [8] implemented a web application for remote sensing flood-induced crop loss assessment using vector tiles, showing that vector tiles are better than raster tiles in terms of bandwidth requirement, loading time and attribute distribution. In addition, the performances of the most popular formats for vector tiles and raster tiles have been compared, and the results show that MapBox Vector (.mvt) is the most efficient encoding format. In addition, some studies have addressed other aspects of vector tiles. Nordan [9] investigated geographical feature topology preservation in vector tile map applications. Two obvious deficiencies of vector tiles are visual discontinuities and graphic conflicts across the borders of tiles when joining geographical features on neighboring tiles and rendering tiles individually with map symbols [2,7]. These flaws can be overcome by applying the devised 'addition' operation to geographical features and map features, respectively [7].

However, efficiency improvement on the client side has been overlooked. The increasing development of data processing, information-integration and information-discovery technologies requires the presentation of spatial entities and geographic phenomena by a more dynamical and efficient means [10–15]. In recent decades, parallel computing has been widely used in GIS research, including land-use modelling [16], vector data visualization [17], geostatistics [18] and polygon intersection [19]. Additionally, the development of CyberGIS [20,21] and spatial cloud computing [22,23] have greatly promoted the application of parallel computing in geospatial applications. Furthermore, E-maps with interactive functions can further improve map availability, strengthen spatial cognition and enhance expressive abilities [24–26]. Interactivity and direct object manipulation are essential functions for spatial data analyses for E-maps [24,25,27–30]. Therefore, it is also important to exploit the potential of vector tiles on the client side to further improve the interactivity of E-maps.

This study proposes an efficient tile-based parallel method for accelerating geographical feature visualization by building computational weight functions of geographical feature visualizations and a workload decomposition strategy. Furthermore, Li et al. [7] proposed the tiled vector data model, which provides a method of avoiding graphical mismatching of symbolized features in map rendering. Thus, a tile-based reconstruction scheme for geographical features is designed to support information queries and spatial analysis based on the tiled vector data model.

This study provides an efficient workload decomposition method for optimizing the parallel visualization of vector tiles on the client side that can be widely adopted and implemented in many applications/platforms without changing the framework of mainstream parallel computing.

The remainder of this paper is organized as follows. We introduce the methods of map symbol organization and symbol representation in the following section. Then, we discuss the tile-based parallel method for geographical feature visualization in section 3. In section 4, a tile-based reconstruction scheme for geographical features is proposed. Section 5 reports the use of the proposed method in a set of experiments and the experimental results. Finally, discussions and conclusions are presented in section 6.

## Map symbol organization and symbol representation

**Fig 1:**
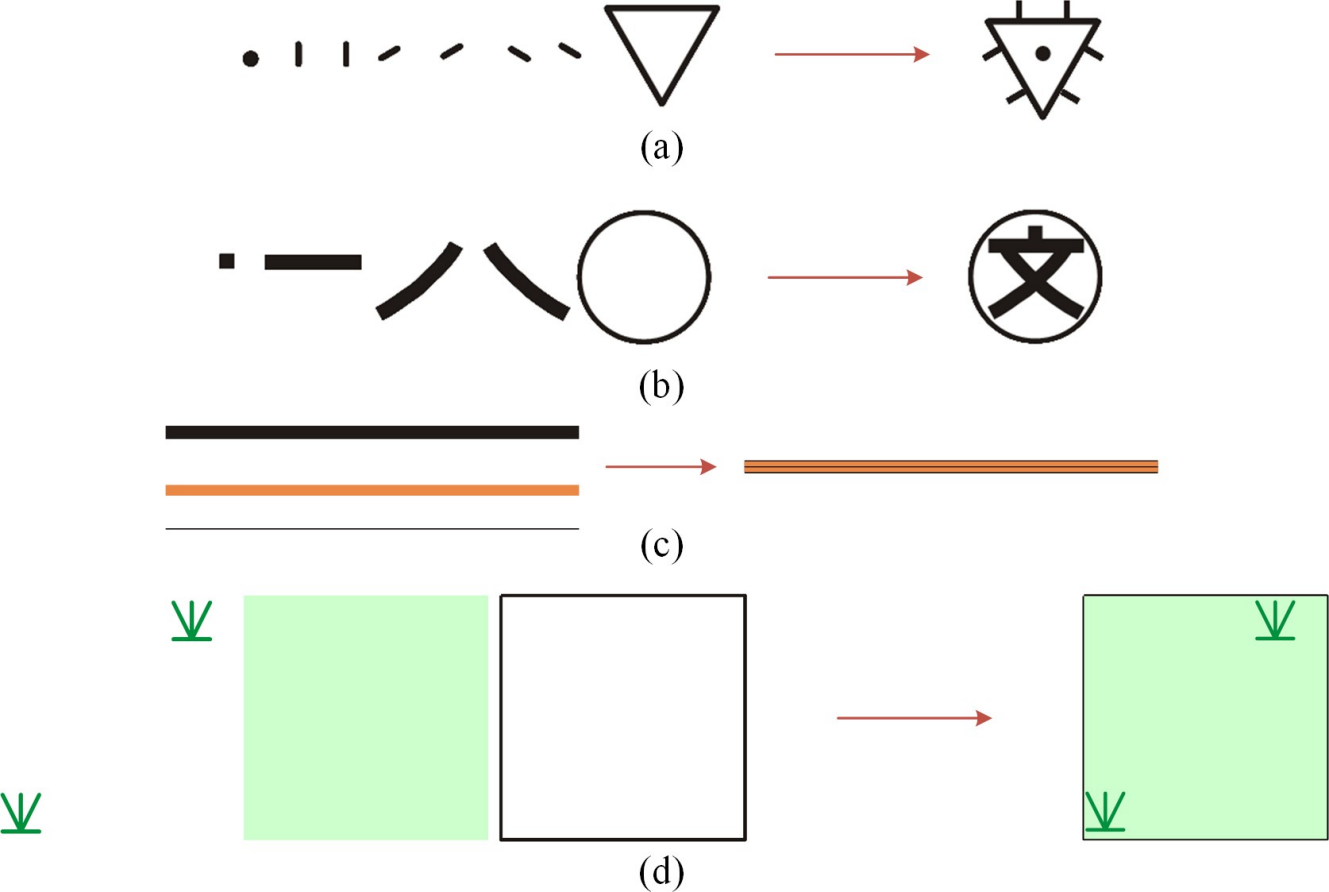
Organization of map symbols.

Map symbols are the essential method of representing geographic phenomena and social phenomena in the real world and can be seen as the language of maps [31,32]. The need for the organization of more complex and visual map symbols is pressing in order to deliver information that can guide and assist the user in solving complex geospatial analysis problems [25,33]. According to previous studies [33–36], this article employs a method based on graphic entities for map symbol organization, and it is especially effective for synthesizing complex symbols [33]. Specifically, the graphic entity is a primitive graphic object, such as a circle, polyline, polygon, rectangle or other graphic object with a line width, color, anchor point and other attributes. Fig 1 shows several examples to illustrate the organization of map symbols.

**Fig 2:**
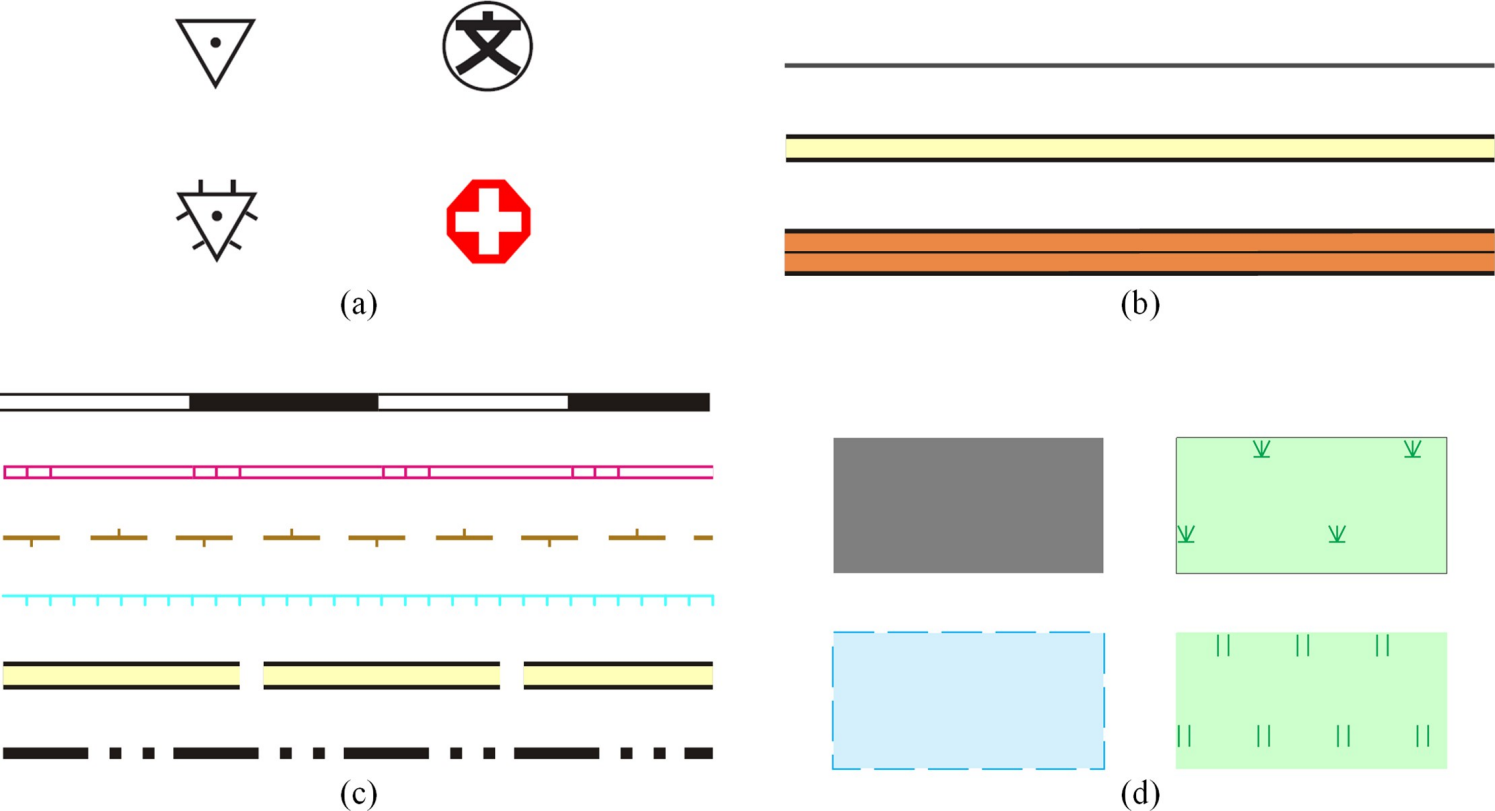
(a) Point feature symbolization results, (b) linear feature symbolization results with simple linear symbols, (c) linear feature symbolization results with complex linear symbols, and (d) area feature symbolization results.

Generally, geographical features can be divided into point features, linear features and area features, and the corresponding symbols are point symbols, linear symbols and area symbols [37]. The symbol representations of different features are also different. Point feature symbolization is a quite simple process that renders the corresponding symbol at the location or coordinate of the feature. The symbolization of linear features is much more complicated than that of point features. When the linear feature corresponds to a simple symbol, the symbolization process renders the graphic entities of the symbol along the coordinates of the linear feature. However, if the corresponding symbol is a complex symbol, graphic entities of the symbol are initially synthesized into a symbol unit, which is then rendered repeatedly along the feature path. Area feature symbolization can be regarded as a merging process of linear feature symbolization and point feature symbolization: if the area symbol contains the boundary symbol, then the symbolization of the boundary can be regarded as the symbolization of a linear feature and the symbolization of the fill pattern in area symbols can be regarded as the symbolization of point features; subsequently, the coordinates of the fill pattern can be calculated by the scan line algorithm or its improved algorithms. Fig 2 shows the symbolization results of geographical features.

## Tile-based parallel method for visualization

Parallel visualization for vector tiles is a typical example of embarrassing parallelism [17][38] because the visualization process of each vector tile is an independent process. Imbalanced workloads for multiple computing units can degrade the performance of parallel spatial computing [39]. Therefore, the performance of vector tile parallel visualization mainly depends on how balanced the workload is distributed onto the computing units. The key to load balancing for vector tile parallel visualization is to estimate and represent the computational weight of each tile efficiently before task allocation. In this section, we first introduce the computational weight of geographical feature visualization, and then, a workload composition strategy for the parallel process of feature visualization is introduced based on the computational weight of feature visualization.

### Computational weight of geographical feature visualization

The visualization process for geographical features consists of three main steps: (1) retrieving the target geographical feature, such as a file or a database, from the dataset; (2) symbolizing the geographical feature; and (3) rendering the symbolization results on the display device [17]. Correspondingly, estimating the computational weight of geographical feature visualization is equivalent to measuring the computational weight of these three steps. For a given geographical feature, the computational weight function (CWF) of geographical feature visualization can be expressed as follows:

$$T(g)=T_{1}(g)+T_{2}(g)+T_{3}(g)$$ (1)

where *T*(*g*) represents the CWF of feature *g* visualization and *T*_1_(*g*), *T*_2_(*g*) and *T*_3_(*g*) represent the CWFs of the three abovementioned steps. To accurately estimate the computational weight of geographical feature visualization, further details of Eq (1) should be considered.

Guo et al. [17] identified the number of vertices of each feature as the key factor for estimating the computing time of geographical feature visualization, thus, this factor can be a good measure for the first step. However, for the remaining two steps, using the number of vertices is not sufficient and only partially fulfills the requirement. For step (2), based on section 2, it is apparent that the computational weights of simple linear symbols and complex linear symbols are different because the computational process of complex linear symbols is much more complicated than that of simple linear symbols. The computational weight of a linear feature with simple symbols can be estimated by the number of vertices of a feature, as demonstrated by Guo et al. [17]. However, when symbolizing a linear feature with complex linear symbols, it is necessary to transform the position of the symbol unit when symbolizing the symbol unit repeatedly along the feature path [37,40]. Notably, position transformation is a computationally intensive process. For the symbolization of area geographical features, the symbolization of boundaries is the same as the symbolization of linear features. In addition, polygon filling requires extra time for area symbols that have fill patterns. Therefore, the result of estimating the computational weight of geographical feature symbolization is biased if only the number of vertices of geographical features is considered and the symbols of the features are overlooked. For step (3), the computational weight of map feature rendering should also be related to the corresponding feature symbol. Section 2 indicates that the map symbols are composed of several graphic entities. Clearly, the more graphic entities a symbol contains, the higher the computational weight of rendering.

Based on the foregoing analysis, the computational weight of step (1) is measured based on the number of vertices of each feature. Therefore, *T*_1_(*g*) can be expressed as follows:

$$T_{1}(g)=O_{1}(n)$$ (2)

where *n* is the number of vertices of the feature, and *O*_1_(⋅)is a linear function.

#### The computational weight of geographical feature symbolization

The computational weight of geographical feature symbolization is more complicated than that of geographical feature retrieval. The weight depends not only on the number of vertices but also on the feature symbol. Furthermore, the calculations of different types of geographical features are different and must be discussed separately. In this article, *T*_2*p*_(*g*)*T*_2*l*_(*g*) and *T*_2*a*_(*g*) are adopted to represent the CWFs of point feature symbolization, linear feature symbolization and area feature symbolization, respectively. The process of point feature symbolization is very simple, and *T*_2*p*_(*g*) can be expressed as follows:

$$T_{2p}\left(g\right)=O_{2p}\left(n\right)=O_{2p}(1)$$ (3)

where *O*_2*p*_(*n*) is a linear function of *n* for a point feature. Without loss of generality, the number of vertices for a point feature is 1.

For a linear feature, different symbols may involve different estimation methods. The estimation method for a simple linear symbol can be the same as that for a point feature:

$$T_{2l}^{s}(g)=O_{2l}(n)$$ (4)

where *O*_2*l*_(*n*) is a linear function of *n* for a linear feature with a simple feature. However, as mentioned in the previous section, when symbolizing a linear feature with complex linear symbols, the number of symbol units certainly affects the computing time of symbolization, and the number of symbol units is equal to the length of the feature divided by the length of the symbol unit. Moreover, the number of graphic entities of the symbol also affects the efficiency of symbolization. Therefore, the CWF of linear feature symbolization with a complex symbol can be expressed as follows:

$$T_{2l}^{c}(g)=O_{2l}^{\prime }(n,\frac{c}{l},m)$$ (5)

where *c*, *m* and *l* represent the feature length, the number of graphic entities and the length of the symbol unit. $O_{2l}^{\prime }(\cdot )$ is a linear function for linear features with complex features, and the parametrized form of $O_{2l}^{\prime }$ is flexible. Overall, the CWF of linear feature symbolization can be expressed as follows:

$$T_{2l}(g)=\left\{ \begin{array}{c} O_{2l}(n),\;simple\;symbol\\ O_{2l}^{\prime }(n,\frac{c}{l},m),\;complex\;symbol \end{array}\right.$$ (6)

Finally, for area features, the symbolization process can be regarded as a combination of linear feature symbolization and point feature symbolization. Therefore, the CWF of area feature symbolization can be expressed as follows:

$$T_{2a}(g)=T_{2l}(g^{\prime })+k\times O_{2p}(1)$$ (7)

where *g*′ is the boundary of *g*, and *k* is the number of fill patterns, which can be estimated by the area of the feature divided by the area of the symbol unit. Therefore, Eq (7) can be rewritten as follows:

$$T_{2a}(g)=T_{2l}(g^{\prime })+\frac{S}{S_{su}}\times O_{2p}(1)$$ (8)

where *S* represents the feature area and *S*_*su*_ denotes the symbol unit area.

Based on the abovementioned considerations, the computational weight of feature symbolization can be expressed as follows:

$$T_{2}(g)=\left\{ \begin{array}{c} O_{2p}(1),\;point\;feature\\ O_{2l}(n),\;linear\;feature\;with\;simple\;symbol\\ O_{2l}^{\prime }(n,\frac{c}{l},m),linear\;feature\;with\;complex\;symbol\\ T_{2l}(g^{\prime })+\frac{S}{S_{su}}\times O_{2p}(1),area\;feature \end{array}\right.$$ (9)

#### The computational weight of geographical feature rendering

Geographical feature rendering involves rendering the symbolization result of geographical features onto a display device. Therefore, this process is closely related to map symbolization. Similarly, *T*_3*p*_(*g*), *T*_3*l*_(*g*) and *T*_3*a*_(*g*) are used to represent the CWFs of point feature rendering, linear feature rendering and area feature rendering.

For a geographical feature with a simple symbol, such as a point symbol or a simple linear symbol, the computational weight of the rendering process is related to the number of feature vertices and the graphical entities contained in the symbol. Therefore, the CWFs of a rendering point feature and a linear feature with a simple linear symbol can be expressed as follows:

$$T_{3p}(g)=m\times O_{3p}(n)=m\times O_{3p}(1)$$ (10)

$$T_{3l}(g)=m\times O_{3l}(n)$$ (11)

where *g* represents the geographical feature, *m* denotes the number of graphic entities of the symbol, *n* is the number of vertices of the feature, *O*_3*p*_(*n*) is a linear function of *n* for the point feature, and *O*_3*l*_(*n*) is a linear function of *n* for a linear feature with a simple symbol. Similar to linear feature symbolization with a complex symbol, the estimation method of linear feature rendering with a complex symbol can be expressed as follows:

$$T_{3l}(g)=O_{3l}^{\prime }(n,\frac{c}{l},m)$$ (12)

where $O_{3l}^{\prime }$ is a linear function for linear rendering with a complex feature. The parameterized form of $O_{3l}^{\prime }$ is flexible; further details are discussed in section 5.

The rendering of an area feature can be divided into the following three steps: (1) rendering the boundary of the area feature which, in essence, is linear feature rendering; (2) rendering a polygon with a single color; and (3) rendering the fill pattern, which can be considered point feature rendering. Therefore, for an area feature *g*, the CWF of area feature symbolization is described as follows:

$$T_{3a}(g)=T_{3l}(g^{\prime })+O_{3a}(n)+\frac{S}{S_{su}}\times T_{3p}(g)$$ (13)

where *g*′ is the boundary of *g*. *O*_3*a*_(*n*) is a linear function of *n* for feature rendering with a simple color, *S* represents the feature area, *S*_*su*_ denotes the symbol unit area, and *S*/*S*_*su*_ is used to calculate the number of fill patterns. Geographical feature computational rendering can be expressed as follows:

$$T_{3}(g)=\left\{ \begin{array}{c} m\times O_{3p}(1),point\;feature\\ m\times O_{3l}(n),linear\;feature\;with\;simple\;symbol\\ O_{3l}^{\prime }(n,\frac{c}{l},m),linear\;feature\;with\;complex\;symbol\\ T_{3l}(g^{\prime })+O_{3a}(n)+\frac{S}{S_{su}}\times T_{3p}(g),area\;feature \end{array}\right.$$ (14)

Details regarding the functions in Eqs (2), (9) and (14) are provided in section 5.

### The computational weight of vector tiles and workload decomposition strategy

The computational weight of a vector tile is the sum of the computational weight of all features. Therefore, the computational weight of a vector tile can be expressed as follows:

$$T(tile)=\sum_{i=1}^{j}T(g_{i})$$ (15)

where *T*(*tile*) is the overall computational weight of the tile, *j* is the number of features contained in the tile and *T*(*g*_*i*_) is the computational weight of the *i*th feature, and the estimation equation is described by Eqs (2), (9) and (14).

Once the computational weight of the tiles is calculated, the workload decomposition is the next key issue. Traditional workload decomposition methods such as vertical decomposition, horizontal decomposition and sequential decomposition have been widely used in spatial domain decomposition [17]. However, geographical features are usually unevenly distributed in space, and regular decomposition methods result in imbalanced workloads for multiple computing units, which degrades the efficiency of parallel visualization. Therefore, a new workload decomposition strategy is needed to evenly decompose the computational weight of geographical feature visualization to each computing unit to achieve load balance. The goal of workload decomposition is to allocate tiles to different computing units according to the computational weight of visualization so that the computational weight of each computing unit is approximately equal when simultaneously visualizing the tiles.

Essentially, the workload decomposition strategy is a *k* -subset problem, which is known to be an NP-hard problem. Because we need to consider the efficiency of the entire vector tile visualization process, the workload decomposition strategy cannot be too complex. The entire procedure of the workload decomposition algorithm is as follows:

**Algorithm:** Vector tile visualization workload decomposition algorithm

**Input**:

    *TT*: the array to store the computation time of each tile visualization

    *TID*: the array to store the ID of the tile corresponding to *TT*

    *k*: the number of computing units

**Output**:

    *R*: the two-dimensional array to store the IDs of the tiles allocated to each computing unit

**Start**:

(1) sort *TT* in descending order, and reorder *TID* corresponding to *TT*

(2) initialize two-dimensional array *R* and define an array *wtSum* of size *k* with 0 to represent the sum of the computational weights of each computing unit

(3) define an integer *minCu* to indicate the index number of the smallest element in array *wtSum* and set *minCu* = 0

(4) **for** *i* = 0 to *TID*.size()– 1 **do**

(5)        add *TID[i]* into array *R[minCu]*

(6)        **for** *j = 0* to *k*-1 **do**

(7)            *wtSum[j]* = sum(*R[j]*)

(8)        **end for**

(9)        *minCu* = the index number of the smallest element in *wtSum*

(10) **end for**

The abovementioned algorithm for workload decomposition may not be the optimal algorithm, but considering the algorithm’s efficiency and ease of implementation, this algorithm is adopted to decompose the workload in this paper.

### Tile-based reconstruction scheme for geographical features

When generating vector tiles, geographical features are clipped into small geometric fragments and stored in different tiles. As the map scale becomes more detailed, features are clipped into more fragments by more tiles. Reconstructing these fragments with the attribute data of adjacent tiles into entire geographical features is crucial for applying vector tiles in spatial analysis and information queries. Furthermore, Li et al. [7] proposed a tiled vector data model (TVDM) that can provide a method for avoiding graphical mismatching of symbolized features in map rendering. Therefore, this study proposes a tiled-based reconstruction scheme for geographical features based on a TVDM to support information queries and spatial analysis based on the tiled vector data model. Geographical feature reconstruction involves geometry data reconstruction and attribute data reconstruction.

### Tiled vector data model

TVDM provides two 'addition' operations on two levels: geographical features and map features. Rendered maps can resolve visual discontinuities and graphic conflicts without weakening the interactivity of vector maps. In a study by Li et al. [7], a series of basic concepts and signs were defined, and the derivation processes of the model were introduced. The TVDMs for geographical features are given as follows:

$$g_{p}=\left\{ \begin{array}{l} g,tile\;contains\;point\;feature.\\ \sigma ,tile\;does\;not\;contain\;point\;features\;and\;map\;features\;intersect\;the\;tile.\\ \emptyset ,map\;feature\;does\;not\;intersect\;the\;tile \end{array}\right.$$ (16)

$$g_{l}=\left\{ \begin{array}{l} \sum_{i=1}^{n}g_{i}^{l}{+}_{G}\Delta g{+}_{G}\sigma ,\;end\;point\;of\;the\;feature\;is\;the\;intersection\;point\;\\ \sigma {+}_{G}\Delta g_{1}{+}_{G}\sum_{i=1}^{n}g_{i}^{l}{+}_{G}\Delta g,\;start\;point\;of\;the\;linear\;feature\;is\;the\;intersection\;point\\ \sigma_{1}{+}_{G}\Delta g_{1}{+}_{G}\sum_{i=1}^{n}g_{i}^{l}{+}_{G}\Delta g_{2}{+}_{G}\sigma_{2},\;linear\;feature\;through\;the\;tile\; \end{array}\right.$$ (17)

$$g_{a}=\left\{ \begin{array}{c} \Delta g\;,fill\;pattern\;is\;color\;filling\\ \Delta g{+}_{G}\sigma ,fill\;pattern\;is\;symbol\;filling \end{array}\right.$$ (18)

where *g*_*p*_, *g*_*l*_ and *g*_*a*_ denote the TVDM of the point feature, linear feature and area feature; *σ* is the expansion feature; *l* represents the length of a symbol unit; +_*G*_ denotes the joining of two geographical features; and ∑ represents the “accumulation” operator for geographical features. In Formula (16), the expansion feature of the point feature is equal to *g*. In Formula (17), for case 1, *σ* is at the end of the linear feature, and *Δg*+_*G*_*σ* = *g*^*λ*^. For case 2, *σ* is located at the head of the line and *σ*+_*G*_Δ*g*_1_ = *g*^*λ*^. For case 3, *σ*_1_ is at the head of the feature and *σ*_2_ is at the end of the feature, and *σ*_1_+_*G*_Δ*g*_1_ = Δ*g*_2_+_*G*_*σ*_2_ = *g*^*λ*^.

### Tile-based reconstruction of geometry data

Tile-based reconstruction of geometry data is a process that involves searching the geometric fragments stored in adjacent tiles and reconstructing them into an entire feature geometry. In general, multiple geographical features are combined into a geographical feature by feature spatial operations, such as union, merge and other tools, in ArcGIS. However, spatial operations are usually time-consuming, which would result in a poor user experience when using E-maps for spatial analysis and information queries. In addition, as indicated by Eqs (16)–(18), the TVDM of each type of geographical feature has the expansion feature *σ*. The expansion feature can resolve visual discontinuities and graphic conflicts, but it produces additional geometric fragments and vertices, which may lead to incorrect spatial analysis results and complicate the process of reconstructing features. Therefore, a rapid method for reconstructing geometry data based on TVDM is required. Zhou et al. [41] proposed a method that uses an index table to store the link relationships between discrete global grids, and it can rapidly find geometric fragments and accurately obtain the vertex series from several grids. With the same fundamental purpose, the tile-based reconstruction of geometry data can adopt this modified method as a reference.

Based on TVDM for geographical features, the tiled feature fragments stored in the tile file include three types of vertices: original vertices, the intersections of the feature and the vector tile boundary and expansion feature vertices. The expansion feature vertices are used only to avoid graphical mismatching of symbolized features during map rendering. It is necessary to filter out these vertices to prevent incorrect spatial analysis results during geometric data reconstruction. Therefore, identifying the expansion feature vertices is the primary task of the data organization stage.

**Fig 3:**
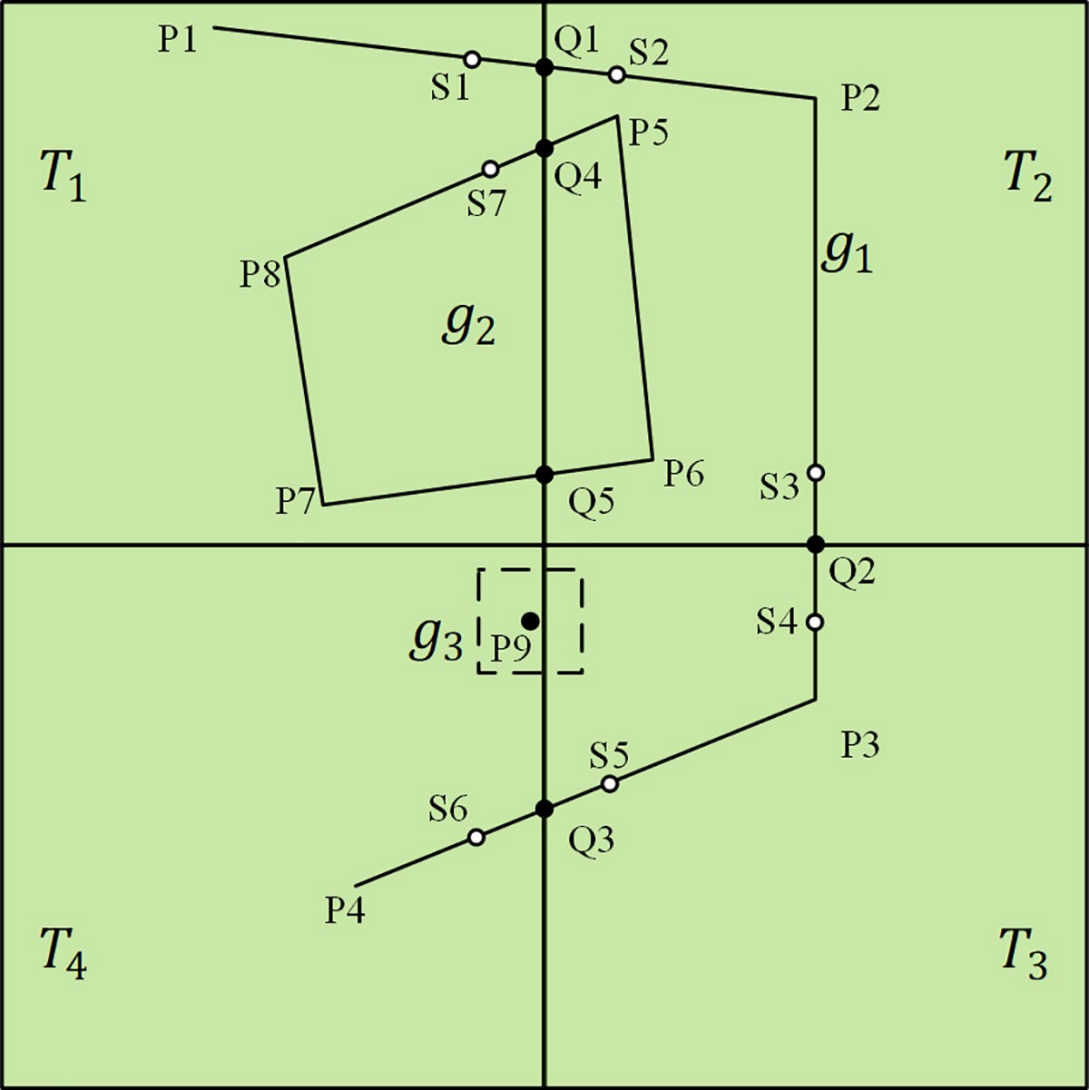
Tile-based reconstruction of geometry data.

Each geographical feature has a unique feature ID (FID). When an entire feature is cut into several geometric fragments by the tile boundary, each fragment saves the original FID as a new attribute called FATHERID (FAID). That is, in multiple tiles, feature fragments with the same FAID can be reconstructed into the original feature geometry. Fig 3 shows an example of geometry data reconstruction. There are three geographical features in the tiles: linear feature *g*_1_ (P1-P2-P3-P4,FID = 1), area feature *g*_2_ (P5-P6-P7-P8-P5,FID = 2) and point feature *g*_3_ (P9,FID = 3). Feature *g*_1_ intersects the tiles at Q1, Q2 and Q3, and feature *g*_2_ intersects the tiles at Q4 and Q5. Based on TVDM, S1–S6 are the expansion feature vertices of feature *g*_1_, S7 and P5 are the expansion feature vertices of feature *g*_2_. The tile file structure based on TVDM is shown in Table 1 where * is the identification sign of the expansion feature vertex.

**Table 1:** Vector tile file structure.

Items Tile Files	FID	FAID	Vertex Series	Other Attributes
T1	2	1	P1-Q1-S2*	…
T1	6	2	Q4-P8-P7-Q5-P5*-Q4	…
T2	3	1	S1*-Q1-P2-Q2-S4*	…
T2	7	2	Q4-S7*-Q5-P6-P5-Q4	…
T3	4	1	S3*-Q2-P3-Q3-S6*	…
T3	8	3	P9*	…
T4	5	1	S5*-Q3-P4	…
T4	9	3	P9	…

In this article, index tables are used to record the link relationships between feature fragments. The items PreTileID (PT), PreFeatureID (PF), NextTileID (NT) and NextFeatureID (NF) are used to record the link relationships between feature fragments in the index tables. Specifically, PT is the tile ID of the tile where the feature fragment before the current feature fragment is located. PF is the FID of the feature fragment before the current feature fragment. NT is the tile ID of the tile where the feature fragment following the current feature fragment is located. NF is the FID of the feature fragment following the current feature fragment. PT, PF, NT, and NF are stored in the index table, and the FAID recorded in each feature fragment contains the orientation and adjacency information of geometric fragments, which can recovery the integrity of the geometry data of the original feature. PT, PF, NT and NF are null if the current feature fragment does not have a previous part or a next part.

Different from the linear features with forward direction, area features have no specific concept of the previous part and the next part, and there may be more than two fragments of homologous geographical feature around an area feature fragments. Therefore, for the area feature, the index table uses only NT and NF items to record the link relationships between feature fragments. Each item can record multiple IDs, the IDs are separated by delimiters, and the IDs order and quantity recorded in NT and NF need to be consistent. The index table structure of Fig 3 is shown in Table 2.

**Table 2:** Index table structure.

Items Index Tables	FID	PT	PF	NT	NF
T1	2	NULL	NULL	T2	3
T1	6	NULL	NULL	T2	7
T2	3	T1	2	T3	4
T2	7	NULL	NULL	T1	6
T3	4	T2	3	T4	5
T4	5	T3	4	NULL	NULL

Based on the tile file structure and index table structure, tile-based reconstruction of geometry data can be realized rapidly and accurately.

### Tile-based reconstruction of attribute data

When a geographical feature is split into several feature fragments by the tile boundaries and stored in different vector tile files, another problem that arises is determining how to address the attribute data of geographical features. Traditionally, raster tile maps store the attribute data on the server side and query attribute data by web services [2]. However, the disadvantage of this approach is that it requires the creation of an additional map service, which can create a complicated system architecture and web services. By comparison, the traditional method addresses attribute data for vector tile maps by assigning the feature’s attribute data to each feature fragment. The advantage of this approach is that all attribute data can be used directly in all feature fragments, and it is easy to implement. Of course, this method inevitably leads to severe information redundancy, which increases the size of each tile file and reduces the transmission efficiency of the files and seriously impacts the user's experience. To avoid information redundancy and improve efficiency, Nordan [9] suggests that only one part of the feature fragment should contain the attribute data while the other parts should store the reference of this information in vector tiles. This article adopts this method of storing the attribute data of features, in which only one feature fragment, called a central fragment, contains attribute data, and the other parts of the feature fragments store the tile ID and FID of the central fragment.

## Experiments and results

A series of experiments were performed on a personal computer (PC) with an Intel i5-4670T CPU (clock at 2.3 GHz, 4 cores/4 threads) with 8 GB of RAM running the Microsoft Windows 10 Education x64 operating system. The map visualization program was implemented using Visual C#.NET based on Microsoft.Net Framework 4.5. The tiled geographical features are organized as the TVDM. The vector tile files are stored in file systems, and the naming rule is based on the rows and columns of corresponding tiles. For example, the name of the tile in row 10 and column 2 is “10_2”. The advantage of the naming rule is its convenience for querying and finding the target tiles effectively and accurately. The parallel algorithm in this article was implemented using Visual C# .Net and Task Parallel Library (TPL). TPL supports program parallelism through the System.Threading.Tasks.Parallel class. This class provides method-based parallel implementations, and the TPL handles the low-level work of the users. Users can easily implement the parallel algorithm by using TPL.

### The coefficients of CWF of geographical feature visualization

**Fig 4:**
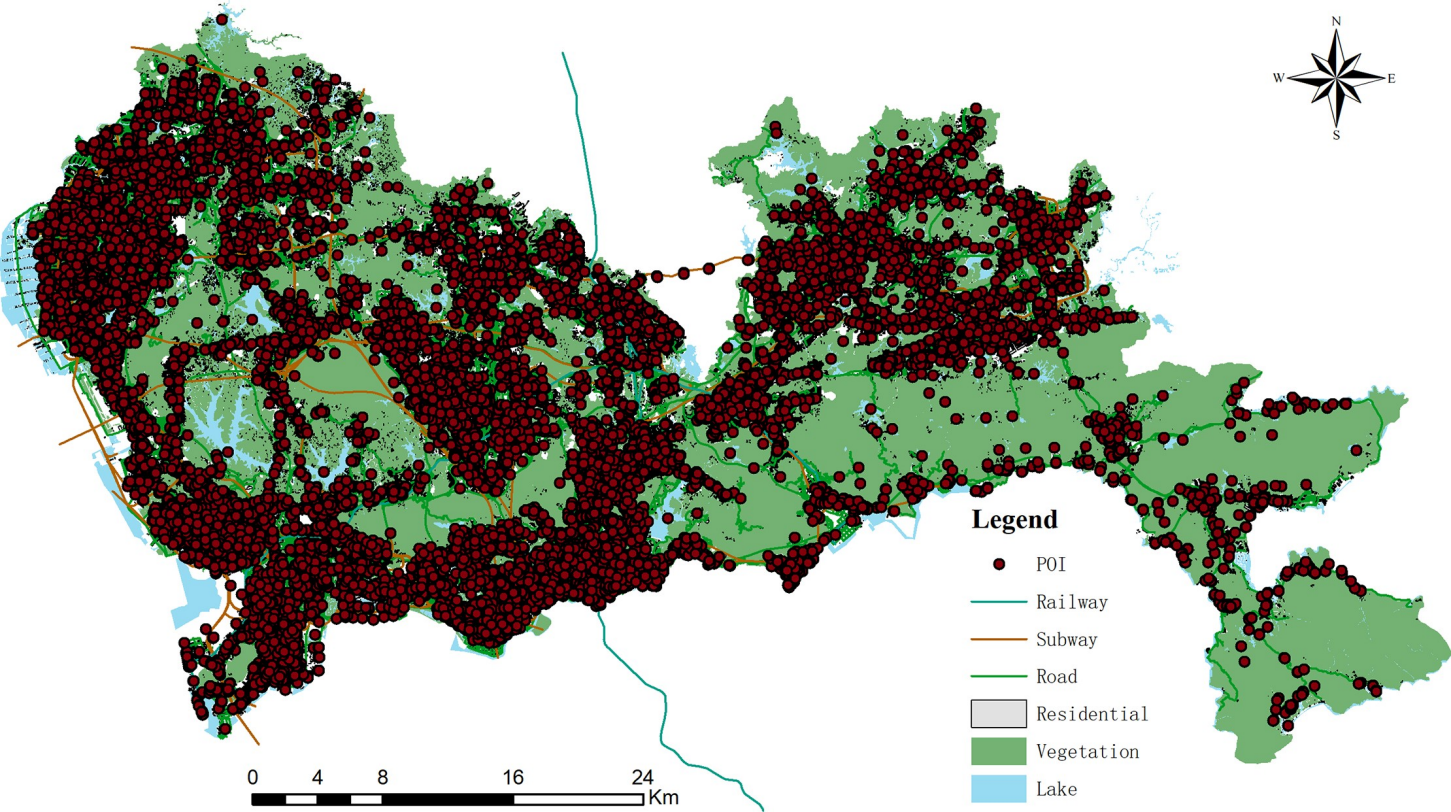
Experimental dataset.

To accurately estimate the computational weight of geographical feature visualization, the specific form and coefficients of the CWFs of three visualization steps must be constructed. A group of real-world vector datasets are used in the experiments as the training dataset to calibrate the CWFs of all three steps. The group of vector datasets were obtained from a 1:10,000 scale electronic map and it consists of a point dataset, a polyline dataset and a polygon dataset. The point dataset is composed of the points of interest (POIs), including 9208 features. The polyline dataset has 11388 road features (the number of vertices of the features ranges from 2 to 1224, the average number of vertices of linear features is 23.6 and the standard deviations is 46.7). And the polygon dataset has 8420 area features (the number of vertices of the features ranges from 3 to 942, the average number of vertices of area features is 36 and the standard deviations is 154.1). Map generalization algorithm cannot effectively solve all the complicated case and some map generalization algorithms have low computational efficiency. Therefore, real-time feature generalization is not appropriate for vector tile. In these experiments, all the features were generalized by Douglas-Peucker algorithm and map generalization is completed before dividing geographical features into vector tiles. The experimental dataset is shown in Fig 4, and the symbols used to calibrate the CWF of geographical feature visualization are shown in Fig 2.

#### The coefficients of CWF of geographical feature retrieval

The CWF of geographical feature retrieval is given in Eq (2). Guo et al. [17] noted that the computing times for geographical feature retrieval are linearly related to the number of vertices. Therefore, Eq (2) can be expressed as follows:

$$T_{1}(g)=\beta_{0}+\beta_{1}x$$ (19)

where the computational weight *T*_1_(*g*) is the computing time of feature retrieval, and *x* is the number of vertices of the feature. These experiments were performed 100 times to obtain the average computing time to reduce the randomness of the experiments. The coefficients of the CWF of geographical feature retrieval estimated from the linear regression model are shown in Table 3.

**Table 3:** Coefficients of CWF of geographical feature retrieval.

Feature Type	Intercept (*β*_0_)	Slope (*β*_1_)	*R*^2^
Point	-	-	-
Line	-0.001	0.0013	0.942
Area	-0.0015	0.0016	0.939

Table 3 does not calibrate the coefficient of CWF of point feature retrieval because the data structure of the point feature is simple; thus, the computing times of point feature retrieval can be neglected. Hence, Eq (2) can be rewritten as follows:

$$T_{1}(g)=\left\{ \begin{array}{c} 0\;,p\mathrm{oint}\;\mathrm{feature}\\ -0.00101+0.00013n,line\;feature\\ -0.00146+0.00016n,area\;feature \end{array}\right.$$ (20)

In addition, the intercept of the CWF is negative because during linear regression, some features have too few nodes, and their independent variables (computing times) are 0, which results in an intercept less than 0 in the regression result. Therefore, when using the CWF to estimate the computing times of feature retrieval, when the estimation result is less than 0, the computing times are set to 0.

#### The coefficients of CWF of geographical feature symbolization

According to Eq (9), the CWF of feature symbolization is more complicated than that of feature retrieval, and the computing times for feature symbolization are still linearly related to the number of vertices for point features and linear features with simple symbols. Therefore, the corresponding coefficients can be calibrated, and the results are presented in Table 4:

**Table 4:** Coefficients of the CWFs of point and linear feature symbolization with simple symbol.

Feature Type	Intercept (*β*_0_)	Slope (*β*_1_)	*R*^2^
Point	-	-	-
Line	-0.0460	0.015	0.9372

The CWF of linear feature symbolization with a complex symbol is related to the number of feature vertices, the feature length divided by the symbol unit length and the number of graphic entities, as shown in Eq (9). However, the specific form of this function is still unknown. Therefore, we assume four functional forms and use them to calibrate the CWFs separately. The calibration functions are as follows:

$$T_{2l}(g)=O_{2l}^{\prime }\left(n,\frac{c}{l},m\right)=O_{2l}^{\prime }(x_{1},x_{2},x_{3})=\left\{ \begin{array}{l} \beta_{0}+\beta_{1}x_{1}x_{3}+\beta_{2}x_{2}x_{3}\;(1)\\ \beta_{0}+\beta_{1}x_{1}x_{3}+\beta_{2}x_{2}\;(2)\\ \beta_{0}+\beta_{1}x_{1}+\beta_{2}x_{2}x_{3}\;(3)\\ \beta_{0}+\beta_{1}x_{1}+\beta_{2}x_{2}+\beta_{3}x_{3}\;(4) \end{array}\right.$$ (21)

where *x*_1_ denotes the number of feature vertices, *x*_2_ represents the feature length divided by the symbol unit length and *x*_3_ is the number of graphic entities of the symbol. The symbols shown in Fig 2(C) are chosen to calibrate the CWF of linear feature symbolization with a complex symbol, and the length of the symbol unit and the number of graphic entities of the chosen complex linear symbol are different. The calibration results of Eq (21) are presented in Table 5.

**Table 5:** Coefficients of the CWF of linear feature symbolization with complex symbol.

Function Forms	Intercept (*β*_0_)	*β*_1_	*β*_2_	*β*_3_	*R*^2^
(1)	-0.063	0.0045	0.0061	-	0.9106
(2)	-0.0658	0.0046	0.0194	-	0.9219
(3)	-0.0787	0.0144	0.0062	-	0.9404
(4)	-0.1692	0.0144	0.0195	0.0303	0.9382

Based on Table 5, the third function has the highest R^2^. Therefore, the CWF of linear feature symbolization with a complex symbol can be expressed as follows:

$$T_{2l}(g)=O_{2l}^{\prime }\left(n,\frac{c}{l},m\right)=-0.0787+0.0144n+0.0062\frac{c∙m}{l}$$ (22)

The CWF of area feature symbolization can be regarded as the set of functions for linear feature symbolization and point feature symbolization. According to Table 4, the computing times of point feature symbolization can be neglected; therefore, Eq (8) can be rewritten as follows:

$$T_{2a}(g)=\left\{ \begin{array}{l} \;-0.0460+0.015n,\;the\;boundary\;symbol\;is\\ \;a\;simple\;symbol\\ \;-0.0787+0.0144n+0.0062\frac{c∙m}{l},the\;boundary\;symbol\\ \;is\;a\;complex\;symbol \end{array}\right.$$ (23)

where n is the number of vertices of the boundary of the area feature, c denotes the length of the boundary of the area feature, *m* denotes the number of graphic entities of the boundary symbol and *l* represents the length of the symbol unit of the boundary symbol. Four types of area symbols are chosen to verify the accuracy of Eq (23). The chosen symbols are shown in Fig 2(D), and the *R*^2^ of the verification result is 0.911.

#### The coefficients of CWF of geographical feature rendering

Similar to the CWF of geographical feature symbolization, the CWF of geographical feature rendering can be rewritten as follows:

$$T_{3}(g)=\left\{ \begin{array}{l} \;m\times (\beta_{0}+\beta_{1}),\;point\;feature\\ m\times {(\beta }_{0}+\beta_{1}∙n),\;linear\;feature\;with\;simple\;symbol\\ \beta_{0}+\beta_{1}∙n+\beta_{2}∙\frac{c∙m}{l},linear\;feature\;with\;complex\;symbol\\ T_{3l}(g^{\prime })+O_{3a}(n)+\frac{S}{S_{su}}\times T_{3p}(g),area\;feature \end{array}\right.$$ (24)

Symbols for calibrating the CWF of geographical feature rendering are shown in Fig 2, and the coefficients of the CWF of geographical feature rendering determined from linear regression are shown in Table 6.

**Table 6:** Coefficients of the CWF of geographical feature rendering.

Feature Type		Intercept (*β*_0_)	*β*_1_	*β*_2_	*R*^2^
Point		0.0023	0.0023	-	0.9483
Linear	Simple Symbol	-0.0031	0.0013	-	0.9476
	Complex Symbol	-0.0468	0.0012	0.0031	0.9601

The area symbols are also used to verify the accuracy of the CWF of area feature rendering, and the *R*^2^ of the verification result is 0.908.

The goodness of fit of all experiments is significant (*R*^2^ > 0.9), which indicates that all coefficient values are significant and that the regressions are effective.

### Accuracy evaluation of the computational weight of vector tiles

**Fig 5:**
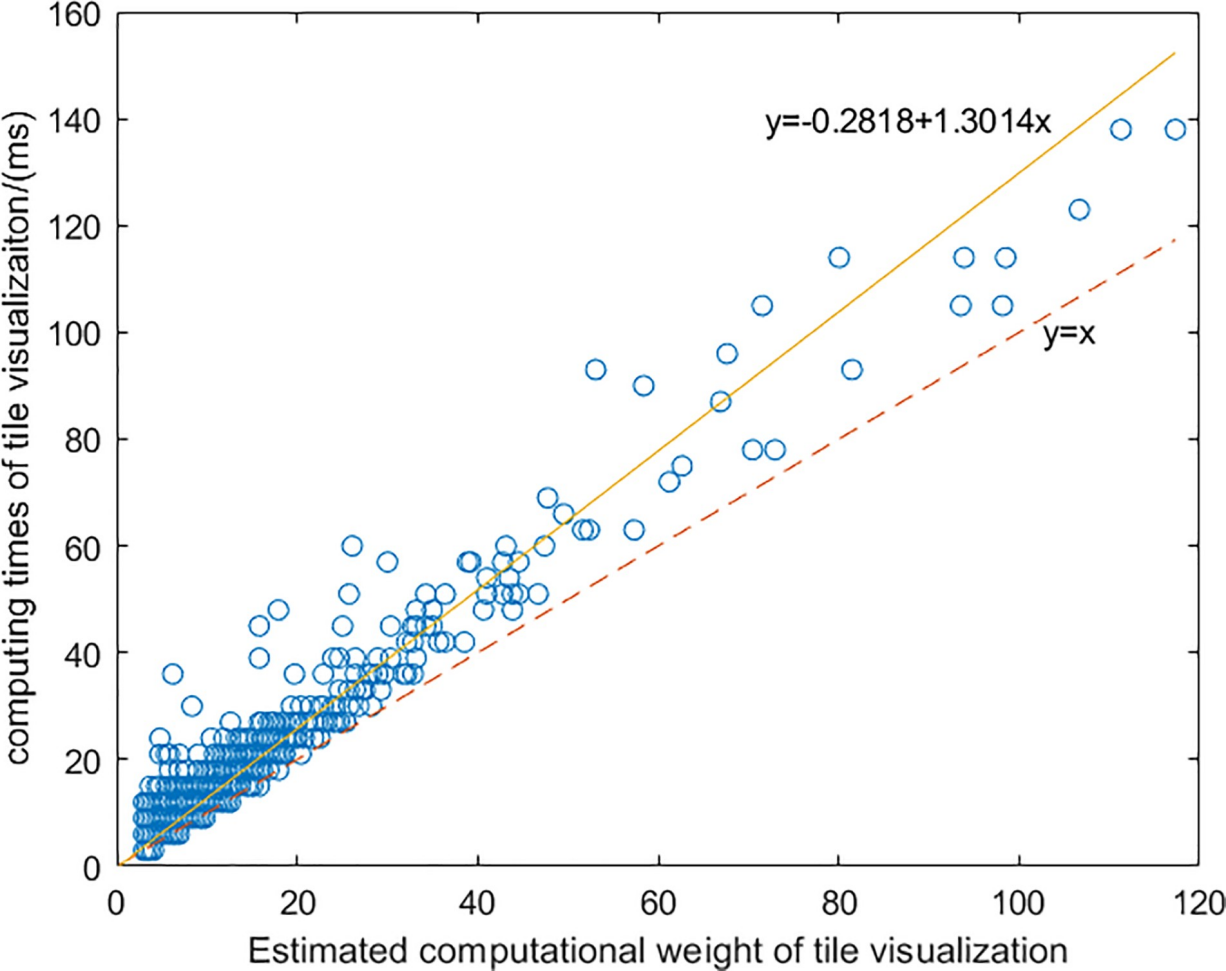
The scatter diagram of the computing times of tile visualization and estimated computation weights.

To assess the accuracy of the computational weight of vector tiles, another group of real-world vector datasets was used. The dataset was at the 1:10000 scale, and vector tiles were generated at the same scale. Like the raster tile map, vector tiles at different scales are generated by geographical features in the corresponding scales. The features stored in the vector tiles were organized based on TVDM, and 9768 tiles were generated. The computational weights of the vector tiles were estimated using the corresponding CWF. Excluding invalid tiles (tiles without geographical feature), we compared the estimated computational weight of vector tiles with the real computing times. As shown in Fig 5, the *R*^2^ of the comparison result is 0.914, which means that the CWF of geographical feature visualization can estimate the computing times of vector tiles.

### Evaluation of parallel visualization efficiency of vector tiles

The map explorer consisted of 24 (6*4) tiles, and each experiment randomly selected 24 tiles from the tiles generated by the abovementioned experiment (excluding invalid tiles) for visualization efficiency evaluation. To evaluate the efficiency, three programs were executed per experiment: (a) a sequential visualization program (using a single thread on one CPU core), (b) a parallel visualization program without a workload decomposition strategy (using 4 threads on 4 cores), (c) a parallel visualization program with the workload decomposition strategy (using 4 threads on 4 cores) provided in section 3. To evaluate the robustness of the workload decomposition strategy proposed in this paper in a multi-core environment, comparison experiments are executed based on both 2 and 4 computing units. We ran 50 comparisons of the experiments, and the results are presented in Tables 7 and 8. In addition, because the vector tile map is a kind of electronic map that needs to be used by a wide variety of users, and the CPUs of most PCs composed of two or four cores, this paper does not verify other multi-core cases.

**Table 7:** Computing times of vector tile visualization with 2 computing units.

Program	Min Time(ms)	Max Time(ms)	Mean Time(ms)
(a)	621	1415	831
(b)	373	882	503
(c)	309	765	418

**Table 8:** Computing times of vector tile visualization with 4 computing units.

Program	Min Time(ms)	Max Time(ms)	Mean Time(ms)
(a)	612	1385	821
(b)	201	482	269
(c)	159	401	219

As shown in Tables 7 and 8, the computing times of vector tile visualization are largely reduced by using parallel computing. Furthermore, the results in Tables 7 and 8 show that the average computing time of program (c) is 18.6% shorter than that of program (b) and that the performance of program (c) achieves near-linear increases in speed compared with that of program (a). Thus, the proposed workload decomposition strategy can further improve the parallel visualization efficiency of vector tiles.

### Experimental results of geographical feature reconstruction

**Fig 6:**
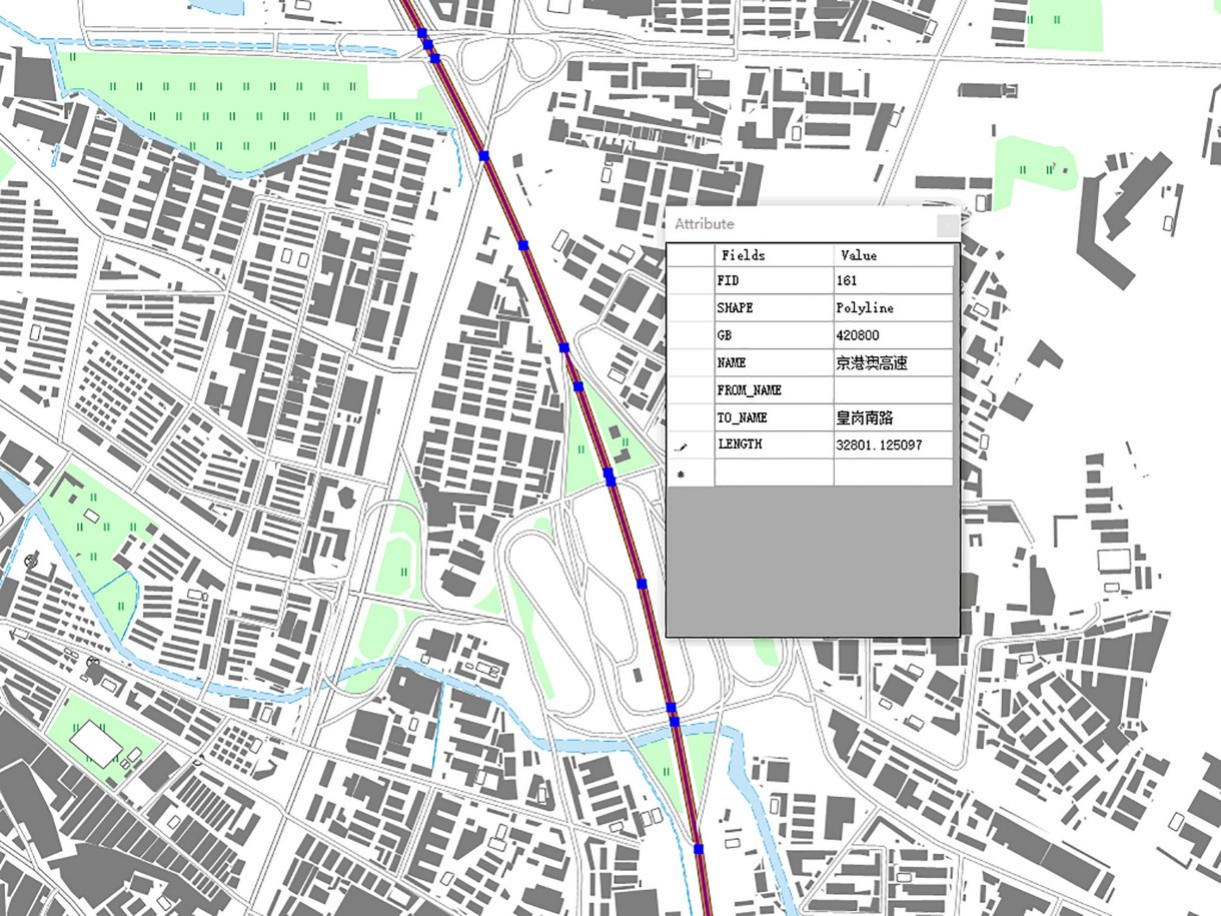
Tile-based geographical feature reconstruction.

Based on the tile-based reconstruction scheme proposed in section 4, users can obtain all features when they select the geographic feature segment in any tile, as shown in Fig 6. The reconstruction features could be used directly for spatial analysis and information query operations. Moreover, as a real-time or near-real-time operation, a quick response is one of the key requirements for user interaction, and the tile-based reconstruction scheme proposed in this article can meet this requirement.

## Conclusion and future work

Vector tiles are a significant technology for representing E-maps. Many studies have considered the transmission efficiency of vector tiles on the server side and have improved transmission efficiency. However, few studies have addressed the improvement of the efficiency on the client side. This article uses parallel computing to improve the visualization efficiency of vector tiles on the client side. Since parallel visualization of vector tiles is a typical example of embarrassing parallelism, the performance mainly depends on estimating the workload and evenly distributing the workload to each computing unit. In this study, we propose using a set of CWFs to estimate the computation weights of geographical feature visualization and the corresponding workload decomposition strategy, which can improve the parallel visualization efficiency of vector tiles on the client side. Moreover, to provide technical support for spatial analysis and information queries, tile-based reconstruction of geographical features is analyzed and discussed.

The conclusions drawn from this study are as follows: (1) The visualization of geographical features is fulfilled in three steps: feature retrieval, feature symbolization and feature rendering. Visualization efficiency is attributed to the geometric structure of geographical features and symbols; a set of CWFs for these three steps is proposed and calibrated by a series of testing experiments. The experimental results show that the CWFs of geographical feature visualization can estimate the computational weight of each tile. The CWFs can be used to estimate both the computational weight of vector tile and the computational weight of other map visualizations. (2) A workload decomposition strategy is developed based on those estimates, and experiments show that the computing times of the parallel visualization of vector tiles are reduced by 18.6% relative to those of common parallel visualization. (3) The tile-based reconstruction method based on TVDM in this study is effective for both geometry data and attribute data. The method proposed for vector tiles demonstrates an intrinsic advantage in terms of interactivity for supporting spatial analysis and information queries, as it avoids many geographical operations used to reconstruct geographical features and render map symbols.

However, the vector tile map in this paper involves only the visualization of geographical features and does not consider feature annotation, which is a necessary element of maps. The annotation of current E-maps is realized through dynamic labelling; therefore, in feature research on vector tile maps, a dynamic labelling algorithm suitable for vector tile maps needs to be designed. Moreover, this study focuses only on computational efficiency on the client side, and overall computational efficiency is improved only by considering all processes on both the server side and the client side and balancing the workload on both sides. In addition, a graphic processing unit (GPU) could be employed in implementation. Incorporating such a GPU will be the subject of our future work. Furthermore, developing the standards of the web vector map tile service is a key point of future research.

## Supporting information

S1 File Experimental dataset. (ZIP)
